# DilatedToothSegNet: Tooth Segmentation Network on 3D Dental Meshes Through Increasing Receptive Vision

**DOI:** 10.1007/s10278-024-01061-6

**Published:** 2024-03-05

**Authors:** Lucas Krenmayr, Reinhold von Schwerin, Daniel Schaudt, Pascal Riedel, Alexander Hafner

**Affiliations:** 1 Cooperative Doctoral Program for Data Science and Analytics, Ulm University and University of Applied Sciences, Ulm, 89075 Germany; 2 Department of Computer Science, University of Applied Sciences, Prittwitzstr. 10, Ulm, 89075 Germany

**Keywords:** 3D dental models, 3D deep learning, Geometric deep learning, Graph neural network, Tooth segmentation

## Abstract

The utilization of advanced intraoral scanners to acquire 3D dental models has gained significant popularity in the fields of dentistry and orthodontics. Accurate segmentation and labeling of teeth on digitized 3D dental surface models are crucial for computer-aided treatment planning. At the same time, manual labeling of these models is a time-consuming task. Recent advances in geometric deep learning have demonstrated remarkable efficiency in surface segmentation when applied to raw 3D models. However, segmentation of the dental surface remains challenging due to the atypical and diverse appearance of the patients’ teeth. Numerous deep learning methods have been proposed to automate dental surface segmentation. Nevertheless, they still show limitations, particularly in cases where teeth are missing or severely misaligned. To overcome these challenges, we introduce a network operator called dilated edge convolution, which enhances the network’s ability to learn additional, more distant features by expanding its receptive field. This leads to improved segmentation results, particularly in complex and challenging cases. To validate the effectiveness of our proposed method, we performed extensive evaluations on the recently published benchmark data set for dental model segmentation Teeth3DS. We compared our approach with several other state-of-the-art methods using a quantitative and qualitative analysis. Through these evaluations, we demonstrate the superiority of our proposed method, showcasing its ability to outperform existing approaches in dental surface segmentation.

## Introduction

The use of three-dimensional (3D) dental models has become increasingly popular in dentistry and orthodontics for diagnosis, treatment planning of tooth misalignments and the fabrication of dental restorations. These 3D dental surface models are obtained by scanning physical impressions (i.e., plaster models) or nowadays, by advanced intraoral scanners (IOSs) that directly reconstruct the digital surface model of the dentition [1]. Precise segmentation and labeling of teeth on such digitized meshes are important for accurate and reliable tooth measurement [2, 3]. Since manually labeling teeth from the dental model is tedious, the development of accurate and automatic 3D tooth segmentation methods for dental models is crucial. However, developing such methods is challenging, since on the one hand the shape and positioning of the teeth is highly dependent on the patient and can deviate greatly from the norm due to tooth misalignments and crowding. On the other hand, the digital scans are influenced by noise and in certain cases do not completely capture the deep intraoral region.

**Fig. 1:**
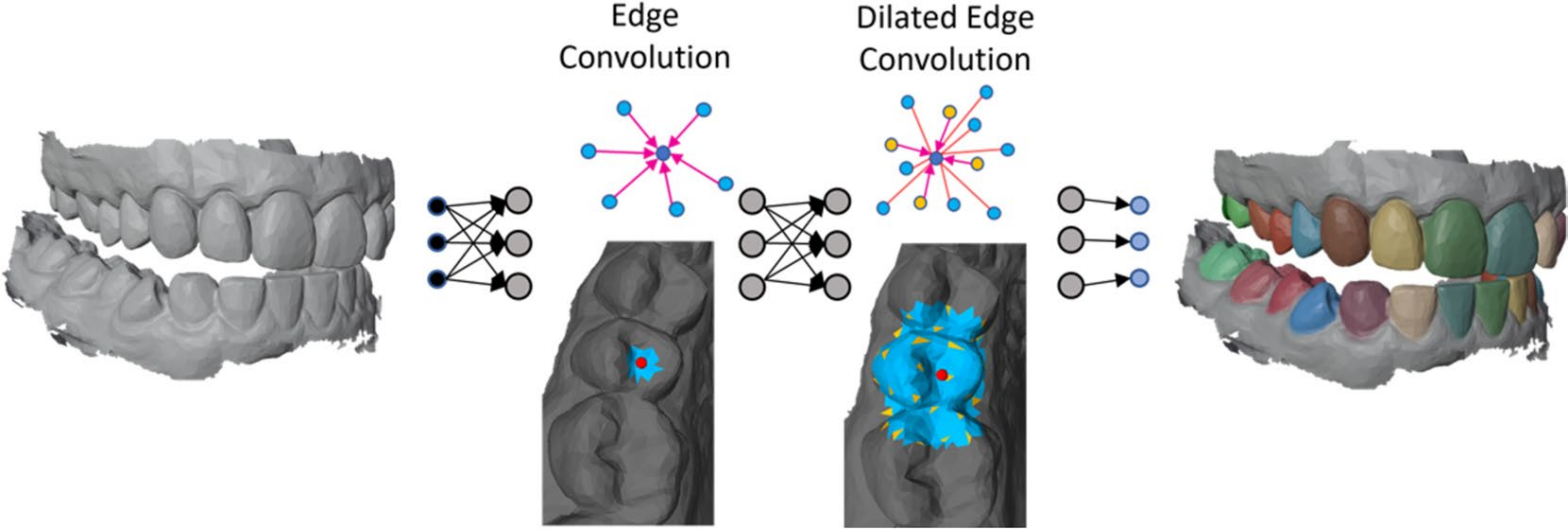
This work introduces a graph neural network for dental model segmentation. The architecture employs edge convolution layers to capture highly informative local features from the input data and introduces dilated edge convolution layers. These integrate more distant features by sampling from the feature space across multiple layers, gradually expanding the receptive field. This enables learning of meaningful local features while capturing broader context

Early approaches often used classical segmentation algorithms such as the watershed approach to segment teeth and gum. However, they are generally not fully automated, as they require user input in the form of starting markers and labeling of segmented regions [4]. With advances in computer vision, researchers have started to utilize deep learning-based methods to address this challenge. These methods are usually applied by either projecting the 3D model into 2D space, which leads to transformation artifacts and loss of information, or by applying deep learning directly to native 3D data representations such as meshes or point clouds. Previous publications [5–8] have explored the application of these approaches for fully automated tooth segmentation. Despite continuous improvement within this domain, all approaches still exhibit certain limitations that hinder their practical applicability, mainly due to insufficient accuracy. Moreover, most of these approaches simplify the problem by assuming the standard case of 14 teeth per maxillary and mandibular, which often does not correspond to the reality of dental anatomy. In practice, patients may deviate from this standard due to missing teeth or the presence of additional wisdom teeth. Additionally, the evaluation of these approaches is frequently performed on proprietary data sets that are not publicly available, making it impossible to reproduce the results. To address this issue, Teeth3DS [9] has been published. This data set provides a publicly available resource for benchmarking semantic segmentation on 3D models, serving as a solid foundation for the present work.

This work introduces a novel feature learning strategy, called dilated edge convolution, which leverages farthest point sampling to emulate an expanding receptive visual field. The concept involves sampling from an enlarging k-nearest-neighbor graph to incorporate more distant features while maintaining a relatively low number of points involved in the edge convolution operation. The objective of this operation is to enhance the accuracy of semantic tooth segmentation, addressing the limitations of existing methods in discriminating between visually similar tooth classes. By increasing the receptive visual field, the aim is to incorporate features of neighboring teeth, utilizing this additional information to deduce the specific tooth class. Evaluations demonstrate that this strategy substantially improves the accuracy of tooth segmentation.

This approach is implemented within a network architecture consisting of dynamic edge convolution and dilated edge convolution layers (see Fig. 1). The effectiveness of our proposed method is supported by multiple experiments and thorough comparisons with state-of-the-art techniques, using the Teeth3DS benchmark data set.

## Background

Deep learning is commonly applied to 3D data analysis, but the structural differences of point clouds or meshes pose challenges due to their lack of grid-like structure. This hinders the direct use of conventional convolutional neural networks (CNNs) popular in computer vision. Earlier approaches aimed to address this challenge by transforming 3D data into a collection of multiple 2D views [10, 11], or by voxelizing data into 3D grids [12, 13]. However, these methods invariably caused the loss of spatial information and introduced transformation artifacts, consequently influencing the performance of the network. To address these limitations, the novel network PointNet emerged [14]. Inspired by spatial transformer networks [15], PointNet enables learning translation-invariant geometric features by employing a series of shared multilayer perceptrons (MLPs) across vertices or faces, along with a symmetric function such as global max-pooling. This approach demonstrated promising results by using the raw surface modeled as a point cloud as input. However, it disregarded the local spatial relationships of 3D shapes, as the network learned features independently for each face. In recent years, various publications have been published aiming to overcome the limitations of PointNet. One prominent example is PointNet++ [16], which introduced a hierarchical network structure that groups points into increasingly larger sets and applies PointNets to learn group-wise geometric features. Although these extensions significantly enhance the results, they often struggle to capture detailed semantic information due to the coarse modeling of local dependencies. A more recent approach is PointNext [17], a further refinement of PointNet++ that introduces residual connections, an inverse bottleneck design, and divisible MLPs to improve the efficiency of the network.

In contrast, graph neural networks have also shown remarkable performance in learning from irregular structures, which has led to the development of several graph-based approaches. Here, 3D data is treated as a graph with points as nodes connected by edges denoting nearest neighbors. To determine the neighbors for each node, the k-nearest neighbor (kNN) algorithm is often employed, utilizing the Euclidean distance between the nodes. A commonly referenced approach in this field is the Dynamic Graph Convolutional Neural Network (DGCNN) [18], which applies edge convolution to the 3D data modeled as a graph. An interesting aspect of this approach is the application of edge convolution on a dynamic graph, which is recomputed after each layer based on the learned feature space. Using the dynamic graph, the network is capable of learning local features by considering not only points that are geometrically close, but also those that are close in the feature space. This enables the network to capture and exploit additional information beyond the input geometric space.

This work extends on these ideas by using dynamic edge convolution to learn highly informative local features which are enriched by more distant features through sampling in multiple layers from an enlarging but sparse k-nearest-neighbor graph. This mimics an increasing receptive field while preserving the ability of the network to learn semantically meaningful local features.

## Related Work

In the field of dental model segmentation, early approaches relied on pre-selected geometric properties, such as mesh curvature [4, 19], or utilized harmonic field-based methods [20] to segment teeth and gum. However, these methods often involved manual intervention and provided only semi-automated segmentation.

To address these limitations, researchers used recent advances in deep learning and proposed fully automated segmentation methods for dental models. For example, Xu et al. [5] introduced a framework that converts dental models into geometric features, which are then transformed into 2D images. These images are then used to train CNNs to classify mesh faces. The network was integrated into a complex pipeline that involved multiple pre- and post-processing steps. Another approach presented by Tian et al. [6] utilized octree partitioning to voxelize the dental model. Subsequently, 3D CNNs were applied to segment the teeth and gum. Although these methods demonstrate promising results in dental model segmentation, they often require additional pre- and post-processing steps, such as feature extraction or conversion of the data into a grid structure. Unfortunately, these additional steps can result in the loss of spatial information and introduce quantization errors.

Recently, the focus on dental mesh segmentation has shifted toward deep learning methods that directly utilize the raw surface data obtained from IOSs. Lian et al. [7, 21] proposed MeshSegNet, an extension of PointNet, which can learn from raw surface data by taking the coordinates of the face vertices and normals as input. By employing multi-scale graph-constrained learning modules, MeshSegNet emulates the hierarchical modeling capability of CNNs to capture multi-scale local geometric context. Furthermore, Zhao et al. [8] presented TSGCNet, a method that also directly accepts face vertices and normals as input. This approach is based on the concept of edge convolution, introduced by DGCNN [18], for semantic surface segmentation. Notably, they introduced the idea of separating the coordinates and normals into two distinct feature streams, to account for their distinct geometric meanings. This separation enables the network to learn more discriminative geometric features by considering the unique characteristics of each feature stream.

Using raw surface data and incorporating advanced deep learning techniques, these methods aim to enhance dental mesh segmentation by exploiting the inherent geometric properties of the data and learning informative representations directly from the surface coordinates and normals.

## Materials and Methods

### Data and Preprocessing

**Fig. 2:**
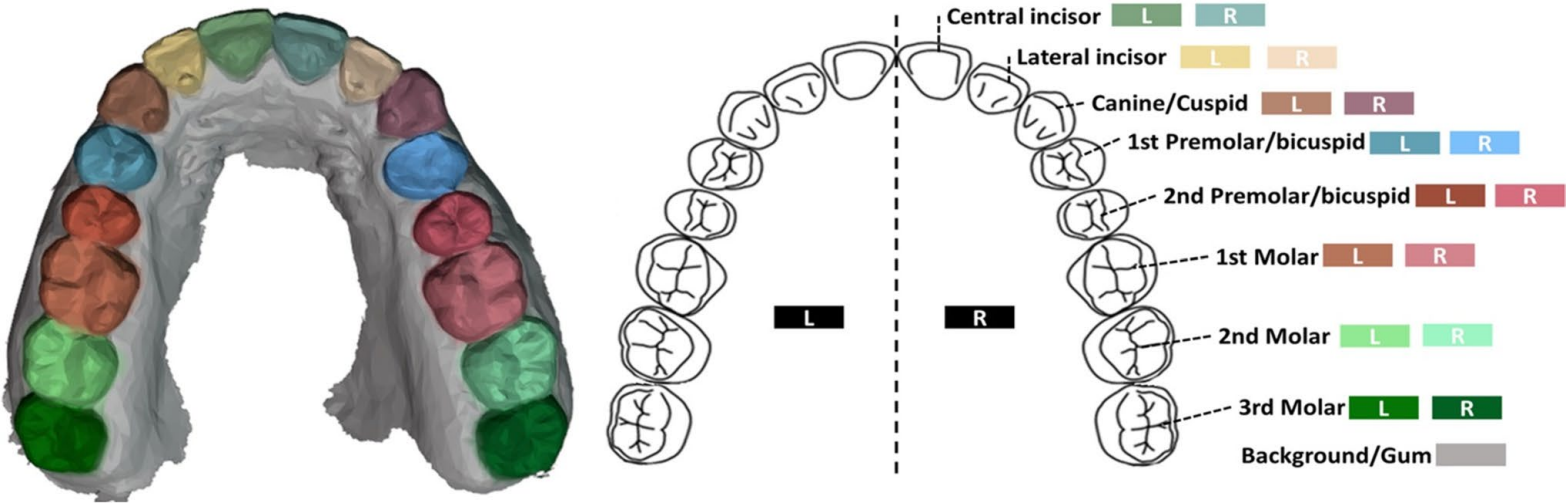
An example of a labeled 3D tooth model captured by an IOS containing all 16 teeth in the corresponding colors (left). An image of a dental arch including the scientific description per tooth and the corresponding color for the left and right quadrants (right)

In this work, we use the benchmark data set Teeth3DS [9] for teeth segmentation and labeling. This data set was recently published through the MICCAI 2022 conference and consists of 1800 unique raw maxillary and mandibular dental surfaces captured directly through an IOS from 900 different patients. The data set contains the IOS scan of the maxillary and mandibular as individual data points once per patient. An example of a labeled dental model and the corresponding coloring encoding with the scientific description per tooth, which is used in further analysis, is shown in Fig. 2. According to the publisher, the data set has been carefully validated by orthodontists and dental surgeons with more than five years of professional experience. Moreover, the data set offers two official test-train splits, each dividing the data into 1200 training instances and 600 test instances. From now on, these splits will be referred to as S1 and S2. Statistical insights into the data set are presented in Table 1. This figure presents an overview of the relative distribution of the number of teeth per case in the entire data set, as well as the number of cases that have an artificial socket. In S1, given that the maxillary and mandibular data points are distinct entities within the data set, there are instances where a patient’s maxilla is present in the training split while their mandible is in the test split (or vice versa). In this case, it is debatable whether the training and testing data are independent, as the maxilla and mandible may develop independently over time but are inherently related as parts of the same patient’s anatomy. However, in S2, the training and test data are independent of each other, as patients do not appear in both subsets. To preserve the fundamental concept of using the data set as a benchmark and to ensure reproducibility of the results, the decision was made to still use the given train/test splits S1 and S2. Notable in both S1 and S2, the distribution of the number of teeth follows a similar pattern, with the standard case of 14 teeth making up more than half of all cases. Furthermore, in S2, the number of cases with sockets exhibits a notable imbalance between the training and test sets. Although only 3.5% of the cases in the training set contain sockets, more than 81% cases contain a socket in the test set. The surface of the dental models is represented as a triangular mesh, which is a type of polygon mesh. A triangular mesh is a 3D surface made up entirely of a set of triangles. Each face of the object is a flat surface formed by connecting three vertices [22]. The original dental surfaces in the data set per data instance (maxillary or mandibular) vary in complexity and roughly contain between 26,000 and 520,000 faces, with an average of approximately 230,000 faces. To facilitate the segmentation process for high-resolution mesh surfaces, a common practice is to down-sample or down-scale the meshes to a standardized size (e.g., as done in previous work such as [7, 8, 21]). In our case, we simplify the meshes to a uniform size of 16,000 faces using the quadric-based edge collapse simplification method [23]. As also done by [8, 21], we apply z-score normalization [24] to the feature vector along each dimension.

**Table 1:** Relative number of teeth over all cases and distribution of cases with and without a socket for split S1 and S2

**Number of Teeth**	**S1**		**S2**	
	**Train**	**Test**	**Train**	**Test**
8	0.08%	-	-	0.17%
9	0.33%	0.50%	0.33%	0.50%
10	1.92%	2.00%	2.17%	1.50%
11	4.08%	4.17%	5.17%	2.00%
12	21.33%	21.00%	27.00%	9.67%
13	10.17%	11.50%	10.92%	10.00%
14	56.33%	55.67%	53.42%	61.50%
15	2.75%	1.50%	0.75%	5.50%
16	3.00%	3.67%	0.25%	9.17%
With Socket	67.83%	75.67%	96.50%	18.33%
Without Socket	32.17%	24.33%	3.50%	81.67%

### Network Architecture

In this work, the aim is to train a network that effectively classifies each face of a dental surface model with *M* faces into one of 17 classes. These classes correspond to whether the face belongs to the gum or one of the 16 teeth. The classes are directly translatable to the FDI notation (dental tooth numbering system). To achieve this, we transform the triangular mesh representation of the dental model into an $M \times 24$ vector, which serves as input to the network. Each face is described by a combination of 3D coordinates of its vertices and the center (12 elements), as well as the normal vectors for each vertex and the face normal itself (12 elements).

The output of the network is an $M \times 17$ matrix, where each row represents the probabilities that the corresponding face belongs to a specific class. This enables the network to provide a classification for each individual face in the mesh.

**Fig. 3:**
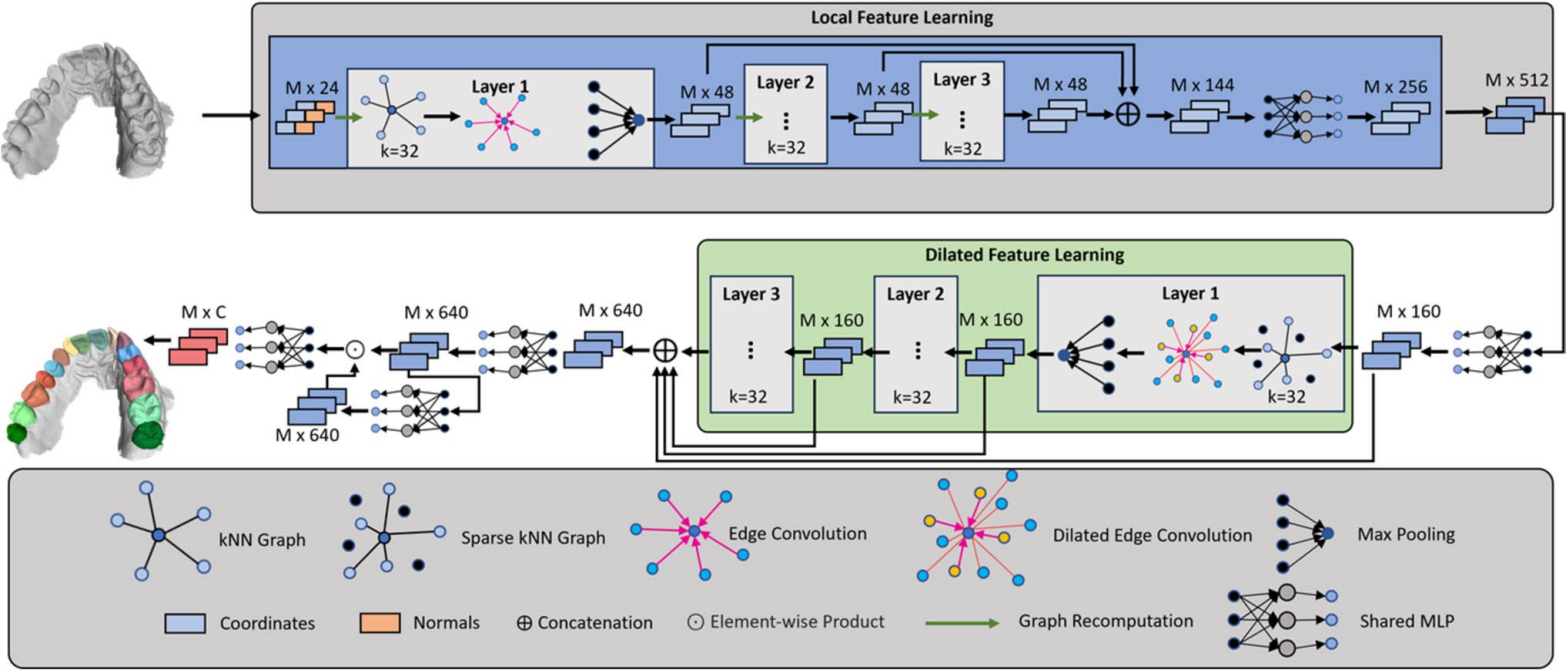
Architecture overview of DilatedToothSegNet: The network processes the coordinates and normals in a local feature learning block utilizing edge convolution layers to learn local features. Local features are used in the dilated feature learning block, which utilizes dilated edge convolution layers to enhance the receptive field and capture broader contextual information. The prediction head consists of point-wise MLPs that estimate the class probabilities per face

As shown in Fig. 3, the network architecture consists of two main blocks: the local and the dilated feature learning block. The local feature learning block employs a stack of dynamic edge convolution layers. These layers are responsible for learning local features for each face of the mesh. Using dynamic edge convolution layers, the network can effectively capture intricate details and patterns at a local level. The dilated feature learning block operates on the learned local features and incorporates a set of dilated edge convolution layers. These layers enhance the features by considering more distant features. This allows the network to take into account information from neighboring faces in a hierarchical manner, enhancing the receptive field and capturing broader contextual information.

Finally, the prediction head is formed using a set of point-wise MLPs. These MLPs output the logits for each face, which are then used to compute the final class probabilities via the softmax activation function. Overall, this network architecture enables effective classification of individual faces in the tooth surface model by utilizing both local and more distant information.

### Local Feature Learning

**Fig. 4:**
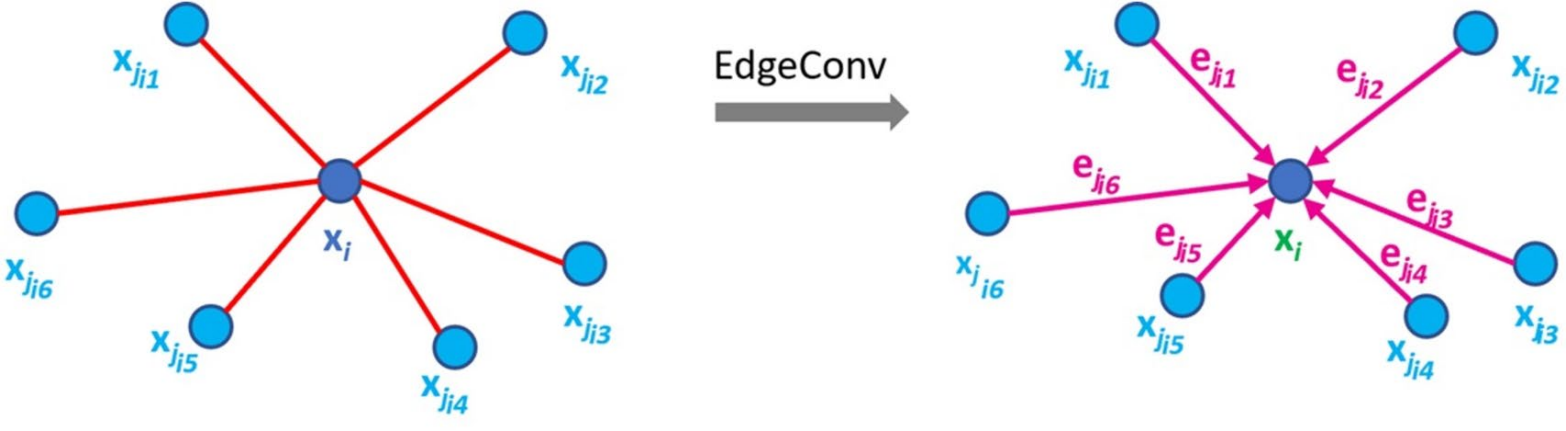
Edge convolution aggregates the edge features associated with all the edges emanating from each connected face (own representation based on [18])

In order to capture local geometric features, we employ the dynamic edge convolution introduced by DGCNN [18], which involves the construction of a dynamic kNN graph and the successive application of edge convolution. The concept of edge convolution is visualized in Fig. 4.

Consider a mesh with *M* faces, denoted by $X = \{x_1, \ldots , x_n\}$. For a face $x_i$ edge convolution learns edge features $e_{ij} = h_\theta (x_i, x_j)$, where $h_\theta$ denotes a parametric non-linear function, which is characterized by a set of learnable parameters. These features describe the relationship between a point or, in this case, a face $x_i$ and its neighbors $x_j$. This is being done by first constructing a kNN graph in each layer by finding the k-nearest neighbors for the *M* faces based on the Euclidean distance in the feature space. For each face $x_i$ we denote the indices of its k-nearest neighbors as $\mathcal {N}\left( i\right)$. Afterward, edge convolution followed by a pooling operation $\boxed { p }$ is applied on each $\mathcal {N}\left( i\right)$ to learn embedded features. The edge convolution is given by Eqs. 1–3 [18]. Here Eq. 1 encodes global information as $x^{g}_i$, Eq. 2 encodes local neighborhood information as $x^{l}_i$ and Eq. 3 combines both global information and local neighborhood information as $x^{c}_i$. $h_\theta$ is implemented as a shared MLP.

1 $$\begin{aligned} x^{g}_i = \underset{\mathrm { j \in \mathcal {N}(i) }}{\boxed { p }} { h_\theta (x_i}) \end{aligned}$$

2 $$\begin{aligned} x^{l}_i \underset{\mathrm { j \in \mathcal {N}(i) }}{\boxed { p }}{ h_\theta ( x_j - x_i }) \end{aligned}$$

3 $$\begin{aligned} x^{c}_i = \underset{\mathrm { j \in \mathcal {N}(i) }}{\boxed { p }}{h_\theta (x_i || x_j - x_i }) \end{aligned}$$

The choice of pooling operation $\boxed {p}$ is an important aspect to consider [25]. The pioneering work of PointNet [14] and DGCNN [18] established max-pooling as a widely adopted choice within this domain. Alternative pooling methods include avg or sum-pooling. Recent work has investigated more sophisticated pooling operations [8, 26] introduce the concept of graph attention pooling, in which a weighted sum-pooling mechanism is employed. This approach involves learning attention weights denoted as $\alpha _i$ and described by Eq. 4, for the neighborhood features of a face $x_i$. These are learned by a lightweight MLP here donated as $h_\sigma$. The input is similar to Eq. 3 the global and local neighborhood features $(x_i || x_j - x_i)$.

4 $$\begin{aligned} \alpha _i = \underset{\mathrm { j \in \mathcal {N}(i) }}{h_\sigma }(x_i || x_j - x_i ) \end{aligned}$$

Following this step, edge convolution is applied in which $\boxed { p }$ is substituted by the sum-pooling after the learned edge features are multiplied element-wise ($\odot$) with the learned attention weights $\alpha _i$.

5 $$\begin{aligned} x^{\alpha }_i = \sum _{\mathrm { j \in \mathcal {N}(i) }}^{ }{\alpha _i \odot {h_\theta (x_i || x_j )}} \end{aligned}$$

TSGCNet [8] also introduces the practice of splitting coordinates and normals into separate streams to allow the network to learn more discriminative geometric features. This involved applying max-pooling in the normals stream and attention pooling in the coordinate stream. However, various training experiments with the described setup using the Teeth3DS data set showed that separating the streams has no positive influence. Consequently, in pursuit of a simpler variant, we abandoned the notion of separating normals and coordinates into separate streams. Instead, we opted for a unified stream employing the edge convolution operation specified in Eq. 3 followed by max-pooling.

### Dilated Feature Learning

Dynamic edge convolution has proven to be a very effective operation in learning local geometric features. In the context of semantic segmentation, it is important to consider not only the features of individual faces but also the surrounding face features. Edge convolution enables this by integrating the features of neighboring faces. Furthermore, the authors of DGCNN [18] claim that dynamic recalculation of the kNN graph in the feature space further enables a decoupling of the neighbor definition from the metric space, allowing to capture semantic characteristics over potentially long distances.

**Fig. 5:**
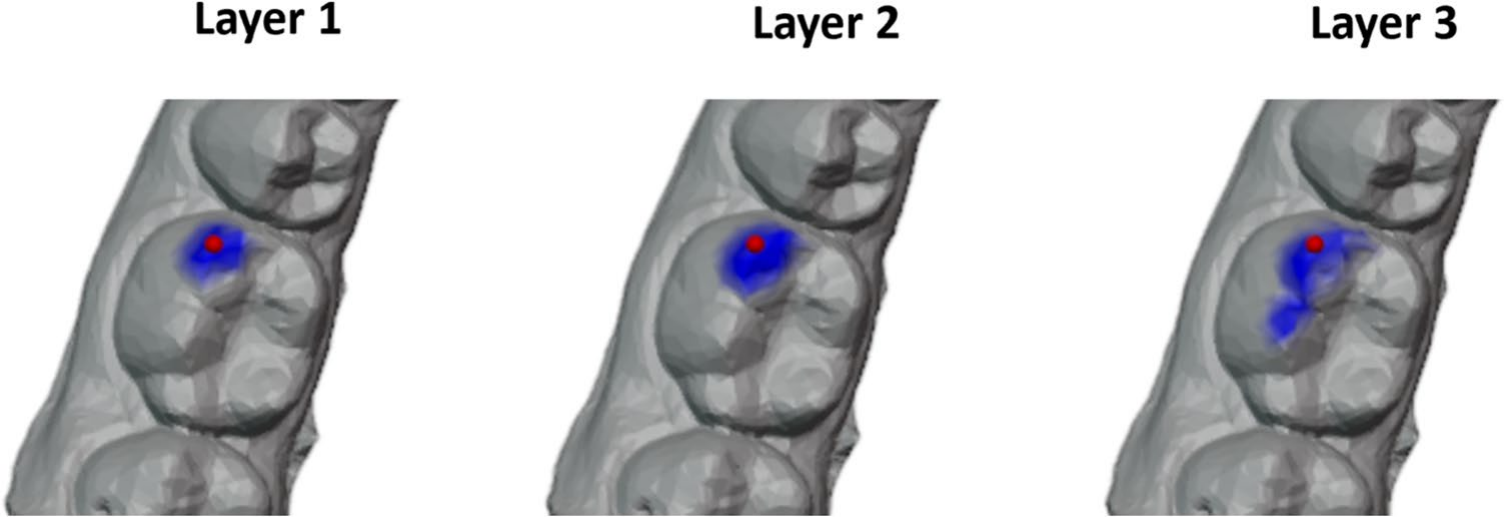
The dynamic kNN graph visualized over three successive layers. The red sphere indicates the focused face and the blue area indicates the nearest neighbors in feature space

**Fig. 6:**
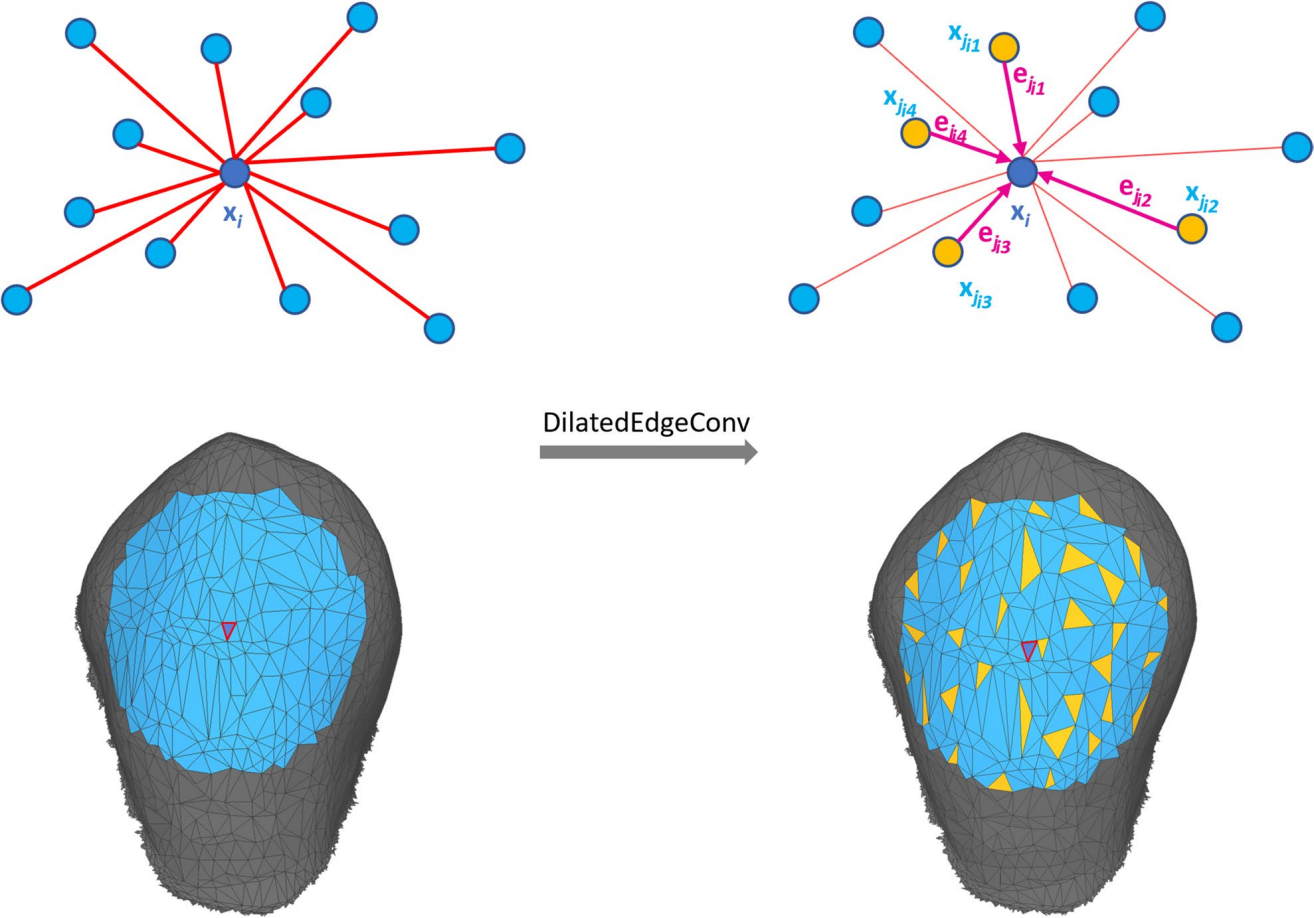
Dilated edge convolution aggregates the edge features associated with all the edges emanating from sampled faces (yellow nodes). The sampling strategy is given by the farthest point sampling from a bigger set of neighbors (blue area). The upper figure presents the concept in a schematic manner, whereas the lower figure demonstrates it on a triangular mesh

For the semantic segmentation of dental models, the decoupling of the neighborhood relation from the metric space is not sufficient to learn features of surrounding teeth. Figure 5 illustrates the progressive detachment of neighboring faces from their Euclidean distance for an example case, as the data propagates through deeper layers of the network. However, even at the third layer, the emphasis remains on local features with no incorporation of features from adjacent teeth. Nevertheless, neighboring teeth from the same tooth group share similar geometric characteristics (e.g., 1^st^, 2^nd^ and 3^rd^ molars). Inclusion of these features in the accurate classification of an individual tooth may prove beneficial. To address this issue, one potential solution could be to expand the network depth by stacking additional layers of dynamic edge convolution. Another solution could be to expand the number of neighbors k considered in the edge convolution process. However, both approaches noticeably increase complexity and demand more computational resources since dynamic recalculation of the kNN graph is a computationally intensive operation. To address this challenge, we present an approach inspired by the dilated CNN in 2D computer vision [27] called dilated edge convolution, which aims to improve the learning of more distant features. The primary objective is to expand the receptive field of the neural network without significantly increasing the number of neighbors involved in the edge convolution. To achieve this, we extend the edge convolution operation through iterative farthest point sampling (FPS) from a larger set of neighboring faces. The following steps outline the procedure, which is also depicted in Fig. 6 and is outlined in Eq. 6:

1. For a given face, denoted as $x_i$, we identify a bigger set of *k* neighboring faces, denoted as $\mathcal {N}_\text {b}\left( i\right)$.
2. From this set of neighbors $\mathcal {N}_\text {b}\left( i\right)$, we use FPS to sample *f* faces, denoted as $\mathcal {N}_\text {s}\left( i\right)$. This sampling technique results in a dilated neighborhood graph that encompasses a larger range compared to the traditional k-nearest neighbor graph while maintaining a fixed number of neighbors. Consequently, we can replace $\mathcal {N}\left( i\right)$ with $\mathcal {N}_\text {s}\left( i\right)$ in the existing edge convolution operation.
3. Finally, we apply edge convolution followed by max-pooling on each $\mathcal {N}_\text {s}\left( i\right)$ to acquire more distant features.

6 $$\begin{aligned} x^{d}_i = \max _{ j \in \mathcal {N}_s(i) }{ }{ h_\theta (x_i || x_j - x_i }) \end{aligned}$$

**Fig. 7:**
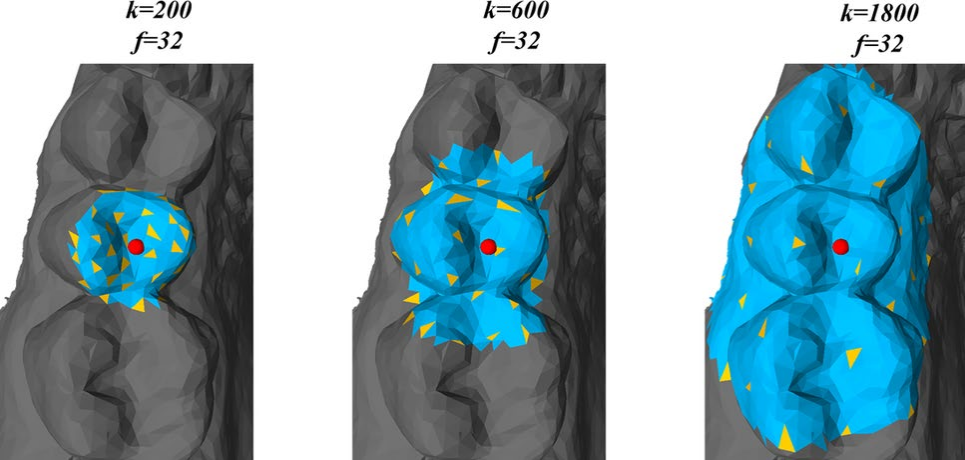
Dilated edge convolution visualized on a dental model for a given face (red sphere) for an increasingly larger sample size. Blue area indicates the larger set of neighbors $\mathcal {N}_\text {b}\left( i\right)$. The yellow faces indicate the sampled faces $\mathcal {N}_\text {s}\left( i\right)$ by FPS

By incorporating the FPS into the edge convolution operation, we can effectively expand the neural network’s receptive field without increasing the number of neighbors involved in the edge convolution. This idea is also demonstrated on a dental model in Fig. 7 for an increasingly larger sample area. In this particular example, the number of neighboring faces *k* in $\mathcal {N}_\text {b}\left( i\right)$ increases, while the number of sampled faces *f* remains the same. Consequently, more distant features are taken into account during the edge convolution operation. However, since this leads to a sparse representation of the surface, it is advantageous to apply this to a feature space that already incorporates neighboring features per face, which is the case after applying edge convolution as described in Section 4.3.

Additionally, it is worth noting that the dilated neighborhood graph is computed on the face coordinates in the metric space and therefore remains static. Consequently, it can be precomputed, thereby saving computational resources.

This operation is integrated in the network as a middle layer after the local edge convolution and operates on the features extracted from the preceding layers.

### Experiment

In this section, we provide a brief description of the competing methods that we used for comparison against our own approach. Additionally, we outline the experimental setup.

#### Competing Methods

To demonstrate the efficacy of our proposed method, we compared it with three state-of-the-art techniques in 3D surface segmentation (PointNet++ [16], PointNext [18] and DGCNN [18]), as well as two state-of-the-art techniques specifically designed for 3D dental model segmentation (MeshSegNet [21] and TSGCNet [8]). The inputs for these methods are briefly described below:

- **PointNet++ and PontNext:** Both networks take as input an $M \times 6$ matrix, where each row represents the 3D coordinates of the face center as well as the normal vector of each face.
- **DGCNN:** Similarly, DGCNN expects an $M \times 6$ matrix as input, where each row contains the 3D coordinates of the face center along with the normal vector of each face.
- **MeshSegNet:** For MeshSegNet, the input is represented by an $M \times 15$ matrix. The first 12 values of each row denote the 3D coordinates of the three vertices of each face and the face center ($3 \times 3 + 1 \times 3$). The last 3 values contain the normal vector of the face. Additionally, small- and large-scale adjacency matrices (AS and AL) serve as input for the graph-constrained learning modules of MeshSegNet.
- **TSGCNet:** TSGCNet expects an $M \times 24$ matrix as input. The first 12 values of each row represent the 3D coordinates of the three vertices of each face and the face center ($3 \times 3 + 1 \times 3$). The last 12 values contain the normal vectors of the three vertices and the face ($3 \times 3 + 1 \times 3$).

For a fair comparison, all methods are trained using the same setup as described in the next section, except for MeshSegNet where the batch size was set to 10. This adjustment was made according to the details provided in the original paper [21], as smaller batch sizes resulted in inadequate convergence.

#### Experiment Setup

The network implementation and experiments were performed using the deep learning framework PyTorch [28]. Training was carried out on two NVIDIA GTX 3090 GPUs. Both the proposed network and the competing networks were trained by minimizing the face-wise cross-entropy loss for a total of 100 epochs, utilizing the Adam optimizer [29]. The distributed data parallelization technique was used for multi-GPU training, with a batch size of 2 per machine, resulting in an effective batch size of 4. The initial learning rate was set to $1e-3$ and underwent a decay of 0.5 every 30 epochs. To quantitatively evaluate the results, we employed the accuracy, mean intersection over union (mIoU), also known as the Jaccard index, and the Dice score as metrics.

Moreover, in the context of segmenting 3D dental models using neural networks, it is common to refine the results obtained by a post-processing step, typically employing the graph-cut method [30]. However, in this study, our primary focus lies on the outcomes generated solely by the neural networks. To allow a direct comparison, no additional post-processing steps were applied to refine the results. Thus, all the results presented in this work represent the direct output of the neural networks without any further post-processing applied.

## Results

In this section, we present the results of a comparison between our proposed method, DilatedToothSegNet, and other advanced techniques for 3D surface segmentation and 3D dental model segmentation. Furthermore, we performed several training experiments to evaluate the effects of the key components employed in our approach.

### Quantitative Evaluation

Table 2 displays the quantitative evaluation results for all competing methods. The table includes the overall accuracy (OA), mean Intersection over Union (mIoU), and Dice score for both splits S1 and S2. Additionally, the table presents the per-class metrics, where the metrics for the same tooth types from the left and right sides are aggregated. Several notable observations can be derived from the results:

- Our proposed method consistently outperforms all other methods in terms of overall accuracy, mIoU, and Dice score.
- In terms of metrics per class, our method achieves superior performance in most cases. However, when it comes to the class of the 3^rd^ molar, MeshSegNet [21] performs slightly better in terms of accuracy. Additionally, for the gum class in S1, TSGCNet [8] shows slightly better accuracy compared to our method. In terms of mIoU and Dice, our method also performs best considering the train/test split 1. However, while all methods achieve insufficient results for the 3^rd^ molar in the train/test split 2, DGCNN performs better.
- Compared to TSGCNet [8], which employs dynamic edge convolution as the primary feature learning technique, our method shows better results. This emphasizes the effectiveness of our proposed dilated edge convolution for additional more distant feature learning.
- Our method also outperforms PoinNext [16] and PointNet++ [16], which rely on iterative grouping and the transformation of points at different scales as their learning strategy. This highlights the effectiveness of utilizing dynamic and dilated edge convolution as a learning strategy for dental models.

**Fig. 8:**
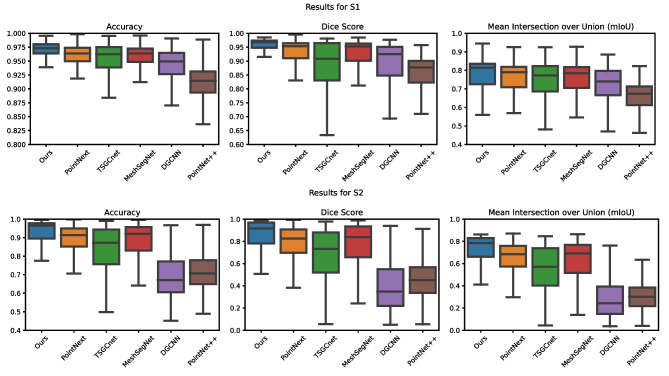
Segmentation results for five competing methods and our method in terms of accuracy, mIoU and Dice score for two different train/test splits as boxplots based on the individual data points in the specific train/test split

Furthermore, the accuracy, mIoU, and Dice score for both S1 and S2 are illustrated in Fig. 8 as box plots, representing the results for each individual data point in the data set. Once again, it is evident that our method outperforms the others and achieves the least deviation. Notable PointNext and MeshSegNet also achieve promising results, followed by TSGCNet. However, TSGCNet tends to exhibit a significant deviation toward low-quality individual results. On the other hand, DGCNN and PointNet++ yield the poorest results. However, there is a noticeable disparity in performance between S1 and S2. In particular, cases without a socket are noticeably worse segmented in S2. This can be explained by the imbalance of cases with and without sockets in S2 as described in Section 4.1. This imbalance leads to a strong tendency during the training toward cases with sockets, which is no longer adequately represented in the evaluation. As a result, the models trained on S2 tend to perform better for cases with sockets, but perform poorly for cases without a socket.

Despite this challenge, our proposed model still produces noticeably better results, demonstrating its ability to generalize effectively and learn features mainly based on the geometric properties of the teeth. This aspect is particularly important in real-world dental scenarios, where different IOSs may be used, leading to variations in socket types and other artifacts.

**Table 2:** Segmentation results for five competing methods and our method in terms of accuracy, mIoU and Dice score for two different train/test splits per tooth type/gum and over all classes (OA). Relevant values in bold

**Model**	**Accuracy**																			
	Split 1										Split 2									
	Gum	3rd M	2nd M	1st M	2nd PM	1st PM	C	LI	CI	OA	Gum	3rd M	2nd M	1st M	2nd PM	1st PM	C	LI	CI	OA
**PointNet++**	93.53	64.45	87.53	87.63	83.64	87.30	85.31	83.87	82.24	89.60	97.60	-	28.71	43.50	47.80	44.16	51.64	55.71	48.54	72.32
**DGCNN**	95.76	65.31	86.38	88.59	88.80	91.69	90.02	90.27	93.62	92.62	95.65	**26**.**54**	41.18	34.46	38.04	37.56	48.61	45.38	57.48	69.53
**MeshSegNet**	96.48	**82**.**44**	92.45	91.54	91.70	93.16	91.98	92.17	92.91	94.08	96.56	13.06	73.83	77.71	82.04	83.39	83.26	79.66	78.42	87.60
**TSGCnet**	**97**.**63**	42.41	85.60	89.13	88.78	91.29	92.08	91.28	92.76	93.57	96.89	4.63	67.81	70.28	70.75	73.00	73.76	67.74	71.06	84.38
**PointNext**	97.20	78.50	92.30	91.17	91.30	93.44	92.18	92.74	92.080	94.22	96.64	14.32	71.31	81.47	85.58	86.33	81.77	79.75	80.09	88.19
**Ours**	97.50	81.85	**92**.**76**	**92**.**90**	**92**.**70**	**95**.**08**	**96**.**00**	**95**.**63**	**95**.**58**	**95**.**41**	**97**.**61**	14.75	**82**.**86**	**86**.**42**	**88**.**00**	**90**.**01**	**88**.**71**	**87**.**31**	**88**.**75**	**91**.**66**

	**mIoU**																			
	Split 1										Split 2									
	Gum	3rd M	2nd M	1st M	2nd PM	1st PM	C	LI	CI	OA	Gum	3rd M	2nd M	1st M	2nd PM	1st PM	C	LI	CI	OA
**PointNet++**	88.59	58.82	78.37	81.09	77.26	78.88	74.32	71.10	70.34	64.25	75.24	-	26.07	37.27	38.58	38.13	41.01	42.02	40.02	33.37
**DGCNN**	92.70	58.69	79.17	83.43	82.94	86.07	84.49	83.33	84.72	70.43	74.07	**18**.**45**	35.14	32.29	31.38	30.61	36.47	37.10	45.43	30.08
**MeshSegNet**	93.86	74.20	86.81	88.11	87.21	88.95	87.57	86.96	87.55	73.78	92.43	12.70	67.40	71.28	70.95	75.71	75.05	71.57	71.25	62.40
**TSGCnet**	94.49	41.23	80.43	85.40	84.83	87.66	87.80	87.21	87.10	72.74	93.25	4.37	60.85	63.50	60.61	64.52	65.69	60.95	62.56	55.30
**PointNext**	94.02	73.17	87.46	88.63	88.06	90.14	88.39	87.83	87.67	74.38	91.35	13.93	65.35	73.20	76.35	78.97	74.98	71.42	70.64	63.43
**Ours**	**95**.**40**	**78**.**57**	**88**.**50**	**90**.**05**	**89**.**95**	**92**.**25**	**91**.**80**	**91**.**70**	**92**.**17**	**76**.**56**	**95**.**62**	13.05	**75**.**71**	**81**.**51**	**82**.**86**	**85**.**60**	**84**.**38**	**82**.**27**	**83**.**28**	**70**.**99**

	**Dice**																			
	Split 1										Split 2									
	Gum	3rd M	2nd M	1st M	2nd PM	1st PM	C	LI	CI	OA	Gum	3rd M	2nd M	1st M	2nd PM	1st PM	C	LI	CI	OA
**PointNet++**	93.88	66.85	86.34	87.26	85.33	87.04	84.22	82.54	81.97	83.12	85.58	-	32.79	47.27	48.78	47.90	52.95	55.30	53.09	47.86
**DGCNN**	96.17	69.04	86.14	88.38	88.53	90.99	90.19	89.81	91.02	86.86	84.73	**25**.**90**	44.51	39.47	38.70	37.10	45.73	46.42	57.25	40.86
**MeshSegNet**	96.80	82.21	91.70	91.03	91.05	92.83	92.27	92.30	92.85	90.07	96.02	18.99	74.98	77.43	78.21	82.25	82.32	79.46	79.74	76.47
**TSGCnet**	97.14	53.67	86.61	89.32	88.98	91.66	92.28	91.94	92.63	86.51	96.46	6.62	68.70	69.95	67.62	71.62	73.18	68.17	70.14	68.23
**PointNext**	96.89	82.05	92.40	91.23	91.36	93.66	92.87	92.87	92.90	90.42	95.39	21.29	74.01	79.79	82.95	85.180	82.53	80.10	79.79	77.29
**Ours**	**97**.**63**	**84**.**66**	**92**.**33**	**92**.**60**	**92**.**85**	**95**.**05**	**95**.**17**	**95**.**19**	**95**.**59**	**92**.**15**	**97**.**75**	20.39	**81**.**26**	**85**.**06**	**86**.**81**	**89**.**34**	**88**.**78**	**87**.**24**	**88**.**02**	**83**.**45**

**Fig. 9:**
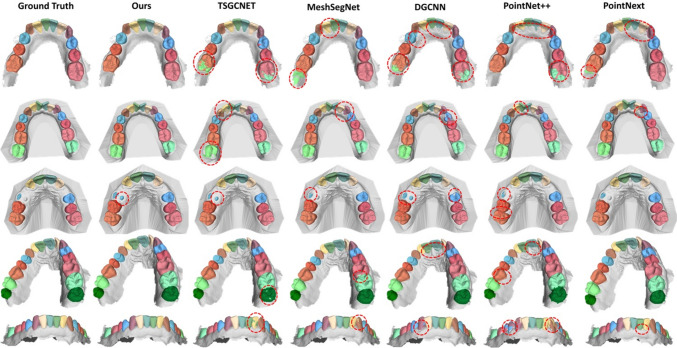
Visualization of five example segmentation results from five competing methods and our method, along with their respective ground truth annotations. From top to bottom: In the first example, the 2^nd^ and 3^rd^ molars are missing. In the second example, all teeth are present except for the wisdom teeth. In the third example, several teeth are missing and the left 1^st^ premolar is barely developed. In the fourth example, all teeth are present, including wisdom teeth. The fifth example shows the front view. The second and third examples also contain a socket

### Qualitative Evaluation

For qualitative assessment, Fig. 9 visually presents the results achieved by the competing methodologies. It includes four distinct examples, each showing unique characteristics. Selected regions with poor segmentation accuracy are highlighted by red circles.

In the first example, the 2^nd^ and 3^rd^ molars are missing. Our method effectively segments each tooth without erroneously identifying any part of the surface as one of the missing teeth. In contrast, the other methods falsely segment certain areas of the gum or the 1st molar as the 2^nd^ molar. This suggests that the other methods primarily focus on the geometry of the teeth, where the 1^st^ and 2^nd^ molars share similarities. However, considering the surrounding area, it becomes evident that it is the 1^st^ molar. The second example represents a common case in which all teeth are present, except the wisdom teeth. In addition, a socket is part of the dental model. Also, the central incisors are severely misaligned. In this case, all methods, except our proposed method, produce misclassified patches in different areas. The third example presents a case where multiple teeth are absent. Additionally, the left 1^st^ premolar exhibits minimal development. In this instance, both TSGCNet and our approach appear to deviate from the ground truth. However, it can be argued that the ground truth in this specific case might be incorrect as it fails to capture all of the surface of the tooth. In the fourth example, all teeth are present, including wisdom teeth. Here, TSGCNet, MeshSegNet, and DGCNN generate reasonable results with only small misclassified patches. However, PointNet++ generates larger misclassified patches and fails to produce clear segmentation boundaries. The fifth example visualizes a case from the front, providing a better view of the segmentation boundaries. In this case, in addition to our method, TSGCNet, MeshSegNet, and DGCNN achieve distinct segmentation boundaries. However, these methods also generate misclassified patches elsewhere. PointNet++ completely fails to provide clear boundaries and generates misclassified patches.

In general, this qualitative assessment further demonstrates that our proposed method excels in delivering clear segmentation boundaries even for cases involving missing or misaligned teeth regardless of whether a socket is present or not.

In addition, we conducted various training experiments that compare the effectiveness of the main components of the presented approach.

### Local Feature Learning Strategy

The local features learning layers employing dynamic edge convolution as described in Section 4.3 play a crucial role in the proposed method, as they capture sensitive local information required for learning highly accurate segmentation boundaries. This work builds up on top of concepts introduced by DGCNN [18] and TSGCnet [8] which utilized the following strategies:

- Dynamic recalculation of the kNN graph after each layer
- Splitting of coordinates and normals into two separate streams
- Using attention pooling for the coordinate and max-pooling for the normals stream

Nevertheless, as our proposed method differs in terms of overall architecture from existing methods and utilizes a different data set, we conducted multiple training experiments with various configurations to assess the effectiveness of the mentioned strategies within the context of our proposed architecture using the Teeth3DS data set.

**Table 3:** Results when using a dynamic versus a static graph, when splitting the coordinates and normals into separate streams and for different pooling methods. Relevant values in bold

**kNN Graph**	**Split Streams**	**Pooling Method**	**Accuracy**			**mIoU**			**Dice score**		
			**S1**	**S2**	**Avg**	**S1**	**S2**	**Avg**	**S1**	**S2**	**Avg**
Dynamic	No	Max	95.41	**91.66**	**93.54**	76.56	**70.99**	**73.78**	92.15	**83.45**	**87.80**
Dynamic	No	Att	95.30	91.34	93.32	76.37	70.33	73.35	91.74	81.96	86.85
Dynamic	Yes	Max N Max C	95.40	91.40	93.40	76.76	70.35	73.56	91.91	83.34	87.62
Dynamic	Yes	Max N Att C	95.42	91.13	93.28	76.65	70.04	73.34	91.60	82.07	86.84
Dynamic	Yes	Att N Att C	95.38	91.30	93.34	76.52	69.71	73.11	91.77	82.93	87.35
Static	No	Max	95.44	91.06	93.25	76.67	69.49	73.08	92.15	82.69	87.42
Static	No	Att	95.08	90.69	92.88	76.19	68.80	72.50	91.32	81.71	86.51
Static	Yes	Max N Max C	**95.47**	91.09	93.28	**76.78**	69.62	73.20	**92.20**	82.95	87.58
Static	Yes	Max N Att C	95.32	90.90	93.11	76.63	69.56	73.10	91.91	81.71	86.81
Static	Yes	Att N Att C	95.37	90.72	93.04	76.64	68.20	72.42	91.88	81.81	86.84

The results are listed in Table 3, based on which the following observations can be derived:

- Max-pooling seems to perform marginally better compared to attention pooling across most configurations, exhibiting slightly higher accuracy, mIoU, and Dice scores for S1 and S2 in several instances.
- Splitting the streams and using a static vs. a dynamic kNN graph show mixed impacts on the results. For the train/test split S1, splitting the streams and having a static graph seems to improve performance, while for S2 it seems that having a single stream with a dynamic graph is the better option. However, in most configurations, the differences are marginal.

It should be noted that the discrepancies found in general are mostly marginal and probably only applicable in the context of this specific data set. Consequently, these discrepancies are not relevant, making it difficult to draw a general conclusion. Therefore, for this paper, the decision was made to consider the optimal average results of both training test splits (S1 and S2). Consequently, a singular stream utilizing max-pooling on a dynamic graph was used.

### Dilated Feature Learning Strategy

As outlined in Section 4.4, we propose the dilated edge convolution operator to enhance the local features acquired through the dynamic edge convolution layers with additional more distant features. To assess the efficacy of these layers, we conducted a set of training experiments using four different configurations and compared the segmentation metrics and visual results. The configurations encompass a spectrum that ranges from the absence of dilated edge convolution layers to the utilization of varying ranges. Additionally, we explored the stacking of multiple layers, progressively increasing in range, as proposed in the final architecture.

**Table 4:** Results when using different settings for the dilated edge convolution layers. Relevant values in bold

**Dilated Edge Conv Layers (Sample Size)**	**Accuracy**		**mIoU**		**Dice score**	
	**S1**	**S2**	**S1**	**S2**	**S1**	**S2**
None	92.59	89.38	70.83	63.21	86.75	81.23
200	93.02	89.50	72.61	64.58	87.93	82.25
600	93.86	89.42	74.27	66.12	88.28	82.06
1800	94.12	90.31	75.21	68.63	90.45	82.77
200, 600, 1800	**95.41**	**91.66**	**76.56**	**70.99**	**92.15**	**83.45**

The configurations and results can be found in Table 4. From the results, the following observations can be derived:

- Utilizing dilated convolution layers in conjunction with dynamic edge convolution layers improves the performance of the network compared to using dynamic edge convolution layers alone.
- Larger sample sizes, leading to a wider receptive field, result in improved performance compared to smaller sample sizes.
- Stacking multiple layers and continuously increasing the sample size further enhance the overall results.

**Fig. 10:**
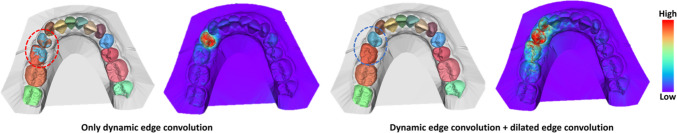
Comparison of using only dynamic edge convolution layers (left) versus also including dilated edge convolution layers (right). Inner left: Shows the predicted labels. Inner right: Shows the class salience map by applying guided backpropagation for the class 1^st^ premolar

The qualitative assessment, as visualized in Fig. 10, confirms these conclusions. The upper section of the figure visualizes the segmentation outcomes of a network that uses only dynamic edge convolution (left) and a network that incorporates dilated edge convolution layers (right). The bottom section illustrates the class salience maps obtained by applying guided backpropagation [31] for the class 1^st^ premolar (L). This example demonstrates that the network generates a more accurate segmentation mask in the latter setup, as opposed to the former. The improved performance can be explained by the additional contextual information provided by the dilated edge convolution layer, which expands the network’s receptive field. The class salience map reveals that the network on the right-hand side utilizes geometric features from farther away. In contrast, on the left-hand side the network focuses primarily on the geometric features of the tooth itself. This discrepancy leads to misclassifications when the geometric properties of two teeth are quite similar. However, by incorporating features from surrounding areas, such misclassifications can be mitigated. In essence, these two scenarios highlight the distinction between predicting the segmentation mask of a particular tooth solely based on the tooth and its immediate surroundings versus considering neighboring teeth and gum tissue.

**Table 5:** Timing Analysis of Dilated Edge Convolution with and without precomputed Indices

**Dilated Edge Conv Layers**	**Average Time in Seconds**	
	Precomputed Index	Without precomputed Index
None	NA	0.0055
1	0.0078	0.0391
2	0.0128	0.0641
3	0.026	0.1291

The time required for a single forward pass through the network using a batch size of 2 with different numbers of layers and whether the indices are precomputed is shown in Table 5. As the number of layers increases, the computational time also increases. When the indices are precomputed, the time gradually increases from 0.0078 s for one layer to 0.026 s for three layers. However, without precomputed indices, the time increases markedly, from 0.0391 s for one layer to 0.1291 s for three layers. These timings are important for both the training and inference phases. Although the computation of the sampling index is time-consuming, the training time can be minimized by precomputing the indices as part of the data preprocessing step. However, the sampling indices are not available during the inference phase. Hence, it is important to consider the time needed to compute these when estimating the overall inference time. However, as the time for a forward pass, which includes calculating the sampling indices, is still well below one second, inference can still be performed relatively fast.

## Discussion

In summary, this study presents several contributions. First, we introduced a novel technique, termed dilated edge convolution, which involves sampling from the feature space with increasing distances through several layers of the network using farthest point sampling. This aims to simulate an expanding field of receptive vision, which ultimately leads to improved tooth segmentation accuracy. Additionally, we proposed a network architecture that integrates dynamic edge convolution layers alongside the newly introduced dilated edge convolution layers. Furthermore, we conducted a comparative analysis of our methods against various state-of-the-art approaches, using the recently published benchmark data set Teeth3DS [9]. Through this analysis, we evaluate the effectiveness of our proposed method. Nevertheless, the following also discusses the limitations of our approach and the data set used. Finally, future research directions are suggested, and our work is positioned within the current research landscape.

### Limitations

#### Extreme Cases and Unbalanced Labels

**Fig. 11:**
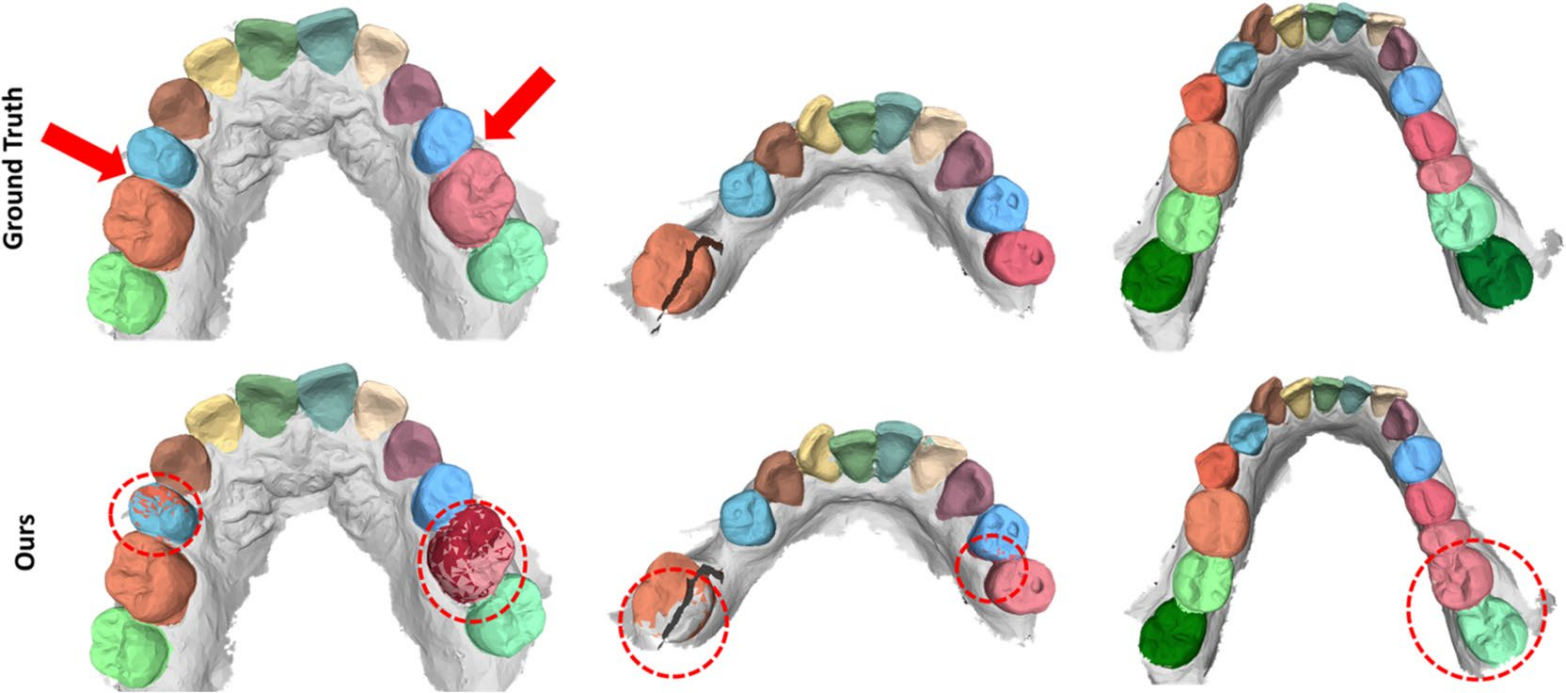
Left: Example where the 2^nd^ premolar is missing on both sides without the presence of a large dental space. The red arrows indicate the position where the missing 1st premolar should be. Center: A challenging case in which several teeth are missing. Right: A case where all 16 teeth are present including both 3^rd^ molar. In the data set the 3^rd^ molar is underrepresented resulting in unbalanced labels. In all these cases, our method fails by yielding some misclassified areas. (see red circles)

Despite achieving improved results in raw dental model surface segmentation, our method exhibits certain limitations that need consideration when it is implemented in practical applications. In Fig. 11, we present three cases in which common limitations are observed. In the first case (left), the 2^nd^ premolar is missing. Unlike most other instances of missing teeth, there is no dental space, making it exceptionally challenging even for human observers to discern the absence of a specific tooth. In this particular case, our network also fails to detect this scenario accurately. On the left side, parts of the 1^st^ premolar are misclassified as the 2^nd^ premolar and on the right side parts of the 1^st^ molar are misclassified as the 2^nd^ premolar. This highlights the inherent difficulty of the task and the wide range of extreme cases that may be encountered.

The second (center) and third (right) cases are representative of the unbalanced labels within the data set. In the second scenario, several teeth are absent, which leads to only 10 teeth being present. On the contrary, in the third scenario, all 16 teeth are present, including the 3^rd^ molars. In both scenarios, our methodology yields misclassified regions. However, this does not occur in all extreme cases. Qualitative analysis reveals instances where our approach adeptly manages these extreme cases. However, it should be noted that such exceptional cases are extremely rare in the data set, making it difficult for the network to learn them and generalize appropriately. Therefore, in future research, it should be considered to address this data imbalance by incorporating additional extreme cases.

#### Data Quality

**Fig. 12:**
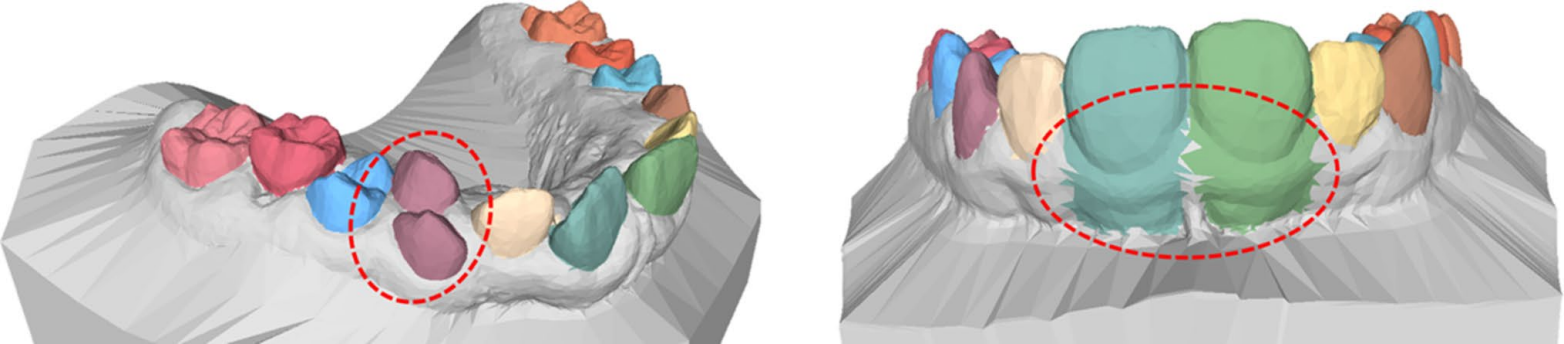
Two examples of falsely labeled data. On the left two teeth are assigned to the same class. On the right a large part of the gum are labeled as tooth

It was observed that there are certain cases within the Teeth3DS data set that are obviously mislabeled. Figure 12 shows two such examples. In one case, two teeth are labeled as belonging to the same class (left), while in the other case, a large part of the gum is mislabeled as part of a tooth (right). Although these cases constitute a relatively small number within the data set, they can still have a negative impact on network training and evaluation. Despite these observations, the decision was made to retain the data set as is and not to manually correct mislabeled cases. This was done to preserve the fundamental concept of using the data set as a benchmark and to ensure reproducibility of the results. Nevertheless, it is recommended that a revised version of the data set should be published. This would contribute to improving the quality of the data set and overall reliability as a benchmark for future research and evaluation purposes.

### Future Work

The dilated edge convolution operation, when used in conjunction with dynamic edge convolution layers, demonstrated promising results. It is worth exploring whether this operation can be applied to a feature space produced by a different backbone network, such as PointNext [32], to potentially achieve even better results.

Furthermore, in the field of dental model segmentation, recent studies have introduced end-to-end segmentation frameworks [32]. These frameworks utilize a multistage segmentation approach, where the first stage involves predicting the location of each individual tooth. In the second stage, the teeth are segmented individually by extracting the region of interest identified in the first stage. Our proposed approach can seamlessly integrate with this multistage approach by serving as the network for the first or second stage.

Moreover, several methods have been proposed to adapt transformer architectures, which have achieved significant success in natural language processing (NLP) tasks, to the field of 3D deep learning [33, 34]. However, the resource-intensive nature of Attention poses a challenge when applying this architecture to high-resolution 3D models, such as dental models. Overcoming this limitation could make the transformer architecture a promising approach for dental model segmentation.

## Conclusion

In this work, we introduced DilatedToothSegNet, a graph neural network designed to automatically segment the surfaces of 3D dental models obtained from IOSs. Building on the work of TSGCNet [8] and DGCNN [18], our approach incorporates the concept of utilizing dynamic edge convolution layers to learn discriminative local geometric features. Additionally, we introduced a dilated edge convolution network operator that effectively learns supplementary more distant features, thereby mitigating misclassified patches and enabling successful segmentation of extreme cases involving missing or misaligned teeth. To assess the performance of DilatedToothSegNet, we performed evaluations on the public benchmark data set Teeth3DS and compared its results with other state-of-the-art methods in the field of 3D point cloud and dental model surface segmentation. The results demonstrate the superiority of our proposed method, highlighting its effectiveness in achieving more accurate and precise segmentation masks compared to existing approaches.

The proposed methodology can be incorporated into a CAD software for treatment planning purposes, automating the otherwise time-intensive segmentation task. Furthermore, the segmentation of dental models serves as the basis for subsequent analysis steps, such as the Bolton analysis [35] for tooth measurement, which therefore requires highly accurate results.

## Data Availability

The data set Teeth3DS used for this paper is available at: github.com/abenhamadou/3DTeethSeg22_challenge

## Code Availability

The code associated with this paper is available at: github.com/LucasKre/dilated_tooth_seg_net
